# 

**Published:** 1830-08

**Authors:** 


### MONTHLY LIST OF MEDICAL BOOKS.

[*Medical Works cannot be entered on this List except a copy be sent for the purpose; the titles of Books having frequently been transmitted to us, as published, which have not appeared for weeks, or even months, after.*]

An Inquiry as to the Expediency of a County Asylum for Pauper Lunatics. Second Edition, with considerable Additions. By William Palmer, d.d. a Magistrate for the Counties of Devon and Somerset.—8vo. pp. 36. Exeter. Hatchard, Piccadilly.

Report of the Managing Committee of the House of Recovery and Fever Hospital in Cork street, Dublin, for one Year, ending 4th January, 1830. With the Medical Report annexed.—8vo. pp. 112. Dublin, 1830.

A concise Treatise on Dislocations and Fractures: being a Selection from the most approved Foreign and English Surgical Authorities, from the Days of Celsus to the present Time. Fourteen Plates.—12mo. pp. 114. London, 1830. Bulcock, Chelsea.

In this little volume, the surgical student will meet with much instructive practical information, in a concise yet perspicuous form.

An Outline of the Sciences of Heat and Electricity. By Thomas Thomson, m.d., Regius Professor of Chemistry in the University of Glasgow; f.r.s. Lond. and Edinb. &c.—8vo. pp. 583. Plates. Baldwin and Cradock, London, 1830.

An Experimental Inquiry into the Structure and Function of the Spleen. By William Dobson. 8vo. pp. 32. Wilson, 1830.

Annali Universali di Medicina. Fasc. di Giugno.

### NOTICE TO CORRESPONDENTS.

*The article from Hastings was immediately inserted in the proper channel, and highly approved of. We never doubted the good taste of our friend, and return him our thanks for this additional proof of his opinion of our own.*

Castigator *lays on the scourge with too coarse a hand. He will perceive that we have referred to the subject in the present Number.*

*The Reports offered by* Medicus *will be very acceptable.*

*Communications have been received from* Dr. *and* Mr. D. Griffin, Dr Alexander Thomson. &c.

*We must decline interfering* editorially *in the affairs of the Western Hospital. If* "An Injured Man" *chooses to forward us a letter with his name subscribed to it, we will insert it.*

*We must entreat our friends to be particular in their designation of this Journal. From the similarity of title which the proprietors of one of our contemporaries have deemed it* expedient *to adopt, Communications intended for us sometimes fall into other hands.*

